# Adaptation strategies: ruminants

**DOI:** 10.1093/af/vfy029

**Published:** 2018-11-10

**Authors:** John B Gaughan, Veerasamy Sejian, Terry L Mader, Frank R Dunshea

**Affiliations:** 1 School of Agriculture and Food Sciences, The University of Queensland, Gatton, Australia; 2 Animal Physiology Division, ICAR-National Institute of Animal Nutrition and Physiology, Bangalore, India; 3 Department of Animal Science, University of Nebraska-Lincoln, Lincoln, NE; 4 Faculty of Veterinary and Agricultural Sciences, The University of Melbourne, Parkville, Australia

**Keywords:** adaptation, constraints, mechanisms, ruminants

**Implications**

- Growing populations and reduced access to arable land mean that animal production systems will either need to intensify and/or produce more from a reducing land and other resource base.
- Variable and unpredictable environmental conditions mean that animal production faces numerous challenges. In addition to climate, these challenges include increased disease risk, increased nutritional deficiencies, and lack of capital to support diversification.
- Predicted changes in climate will impose selection pressures on traits important for biological fitness (and production).
- Genetic adaptation is important for the future of livestock systems. Animal adaptation involves trade-offs, which must be considered when selecting animals for use in breeding programs.

## Underlying Problems

Why do animals need to adapt? What are the issues that need to be addressed (e.g., poor fertility, nutritional challenges)? These are the important questions livestock producers and animal scientists face. Animal adaptation is a function of a number of intertwined factors (i.e., animal × management × resources). Animal adaptability is as much about the animal as it is about the adaptability of caretakers and their use of available resources (e.g., land, feed, water, and capital). Any discussion about animal adaptability needs to encompass all of the factors that will either enhance or reduce adaptability (Gaughan and Cawdell-Smith, 2017). Furthermore, short-term and long-term strategies to enhance adaptation need to be considered.

Broadly, adaptation is a nongenetic (short-term or phenotypic) and genetic (long-term or generational) response to a challenge (stressor). Nongenetic responses to a stressor may be short term such as reduced feed intake and increased respiration rates when exposed to high ambient temperature. However, short-term responses also have a genetic basis with some animals better able to cope than others when exposed to the same stressors. Many management strategies are short-term responses to acute challenges such as provision of shade and dietary manipulation. These reduce the challenge but don’t lead to genetic change.

Productivity gains via targeted trait selection of ruminants are well documented. However, selection of animals for high levels of production has increased animal susceptibility to environmental challenges. For example, it is well accepted that high producing dairy cows are more susceptible to heat stress than low producing cows. Using lower production cows could reduce heat stress, lower milk output, and lower input costs. However, there would be a concentration of maintenance costs with a reduction in efficiency and increased greenhouse gas intensity. Optimum animal production is easiest, but not necessarily the most economical, to achieve under controlled environmental conditions, which is more often seen in nonruminant compared with ruminant production systems.

The challenges are many and do not always have a direct effect on animal performance. For example, chronic exposure to hot conditions may result in poorer pasture quantity and quality leading to poorer nutrition and nutritional outcomes which results in reproductive failure, poor growth, and increased disease risks. In this arena, animal adaptation is not necessarily paramount since it is more about getting nutrition correct and thus a whole farm approach is required (Thamo et al., 2017). The challenges are, to determine if there is a need for adaptation, a need for improved animal management (i.e., management and resource adaptation) or both.

## Adaptation to What?

Enhancing animal adaptation will only work if other aspects of their environment/management are also adapted. For example, developing a heat tolerant bovine is of little value if there is insufficient feed and water to allow the genetic expression of the desired traits or if the productivity of these animals is extremely low. It is important that we understand that animal responses to a given set of stressors may change over time because the animal is adjusting to that stressor or challenge. While it is possible that acclimatization or adaptation may alleviate a stress response, the animal’s performance (milk production, growth rate, fertility) may not return to the prestress levels. This is the conundrum or trade-off that livestock producers and animal breeders face. Adaptation is often at the expense of performance, and survivability is often better in “low” performance animals because their input needs (especially feed) and internal heat production are not as great (Gaughan and Cawdell-Smith, 2017). Stress tolerant animals tend to have lower productivity because they are adapted to the conditions. It was suggested by Colditz and Hine (2016) that there should be an increased focus on breeding and managing animals for improved resilience to applied stressors. They stated that husbandry practices that incorporate physical and social stressors plus interactions with humans could be used to characterize resilient phenotypes to a given set of challenges.


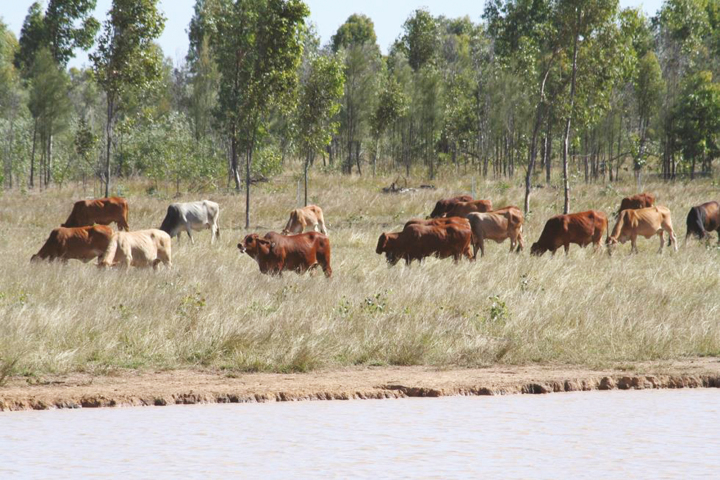


Key to our understanding of animal responses to a stressor(s), and indeed their ability to adapt, is to define the stressor(s), and define what we want the animals to adapt too. People often talk about the negative impacts of high ambient temperature on animal performance. However, animals are rarely exposed to a single stressor. For example, Sejian et al. (2013) discussed the effects of multiple stressors on sheep and concluded that the cumulative effects of excessive heat load, poor nutrition and the need to walk long distances to source feed and water compromised production and reproduction in Malpura ewes (an adapted native breed of semi-arid tropical regions in India). While a single stressor may be important, the cumulative effects of multiple stressors are significant, and some of these may be multiplicative rather than additive.

## Adaptation Strategies

The strategies used to sustain ruminant production can be broadly classified as adaptation (e.g., developing tolerant breeds, improving water access, improved pasture species), mitigation/amelioration (e.g., nutritional interventions, manipulation of the rumen eco-system, provision of shade, housing, fans, and sprinklers; Table 1).

**Table 1.** Livestock adaptation strategies

Parameters for adaptation	Livestock adaptation strategies
Production adjustments	• Changes in quantity and timing of precipitation may shift timing of breeding, feed availability and water availability, and species mix
Genetics	• Identify existing breeds, especially “indigenous breeds” that are already adapted to climatic and nutritional stress • Identify the genes responsible for reducing stress • Functional genomics • Breed improvement through cross-breeding, and incorporation of “stress” tolerant genes
Science and technology	• Understanding of the impacts that environmental and nutritional stress has on animal performance and from this develop new breeds, improve animal health, and improve performance • Enhance soil and water management, develop drought and heat tolerant plants, improved grazing strategies, reduce runoff, and enhance soil fertility • Determine the climatic thresholds that lead to excessive heat load between breeds and species
Animal management systems	• Ensure that there is adequate shade and water to reduce heat stress • Ensure that housing is engineered to reduce the impact of high temperature/humidity • Ensure that housing is cost-effective • Reduce livestock numbers—match animal numbers to available resources • Change livestock species (goats instead of cattle) • Changing land use (land tenure) • Improved management of grazing lands (reduce over grazing and land degradation)
Capacity building	• Training in agro-ecological technologies and practices • Access to finance, energy, transport • Government policy

Adapted from Sejian et al. (2015), Gienapp et al. (2008), Thornton et al. (2009), Zhang et al. (2017), and Escarcha et al. (2018).

In a review of mitigation and adaptation needs of livestock, Zhang et al. (2017) stated that in general livestock producers have adapted to climate change by (1) shifting from cropping to grazing; (2) adopting mixed crop-livestock systems; and (3) decreasing stocking rates and/or herd sizes. However, they concluded by saying that the responses do not necessarily overcome all adverse effects that will be encountered.

There are no universal strategies. Some strategies may have global applicability, others regional, and others at a farm level. Of some concern is that there does not appear to have been any systematic global reviews on how the livestock sector is affected by and adapts to climate change (Escarcha et al., 2018).


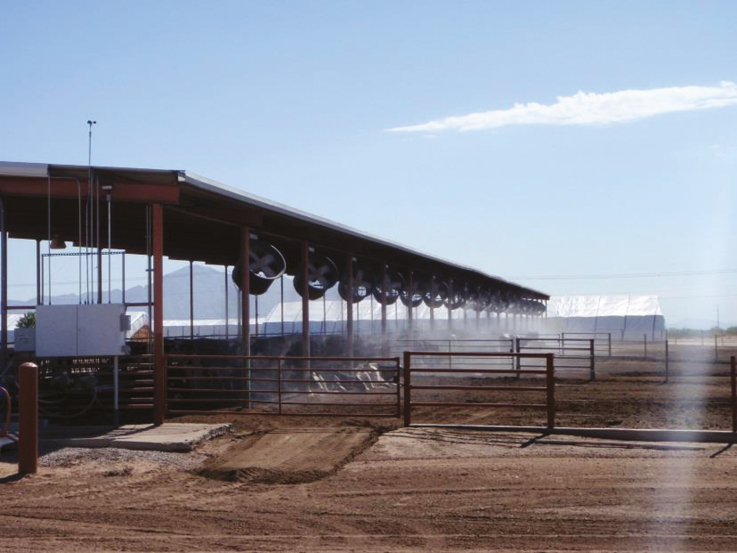


## Constraints to Adaptation

A recent review by Escarcha et al. (2018) listed a number of factors which are likely to constrain adaptation strategies. These include a lack of information at the systems level; lack of adequate research especially in Asia and South America; the fact that capacity building is highly dependent on government and other institutions; pastoral systems especially communal land tenure systems; limited access to natural, capital, and labor resources; poor market infrastructure and organization. Other areas constraining adaptation are a lack of trust in the science of climate change and the many unknowns regarding how climate change will impact on livestock systems.

## Mechanisms of Adaptation

### Morphological adaptation

Morphological adaptations include short and thin hair, light hair color, lightly pigmented skin, higher density of sweat glands, slender legs, and less subcutaneous fat. The coat is the primary protective layer against the direct effects of solar radiation. Fanta (2017) reported that cows with light coat colors in tropical regions reflect solar radiation; thereby protecting the animal from the adverse effects of solar radiation. Whereas cattle with a dark coat color will absorb more solar radiation which increases their heat load. Cattle that are adapted to arid regions possess smooth, short and thin hair (slick hair gene) which enhances heat dissipation. Sweating allows animals to cope in hot climates. In cattle, thermo-tolerance is directly associated with the sweat gland density and sweating rate. Consequently, animals in hot regions maintain sweat glands with higher diameter, volume, perimeter, and density. In addition, cattle breeds in tropical regions tend to have a smaller body size as compared to temperate breeds (Sejian et al., 2018).


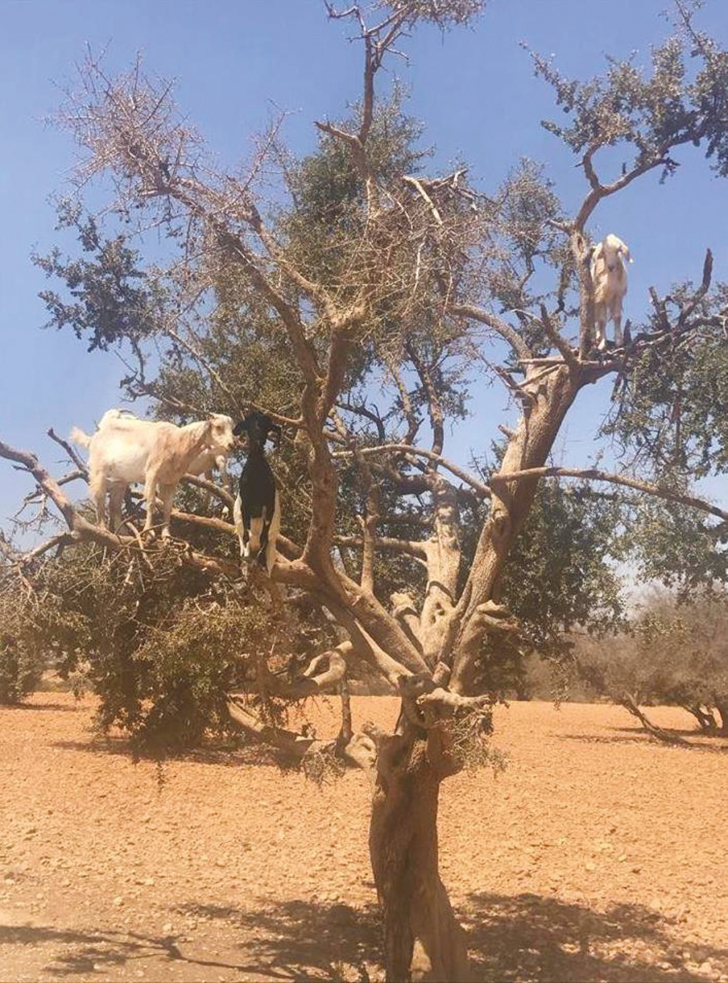


Sheep with light coat colors, which are sleek and shiny, reflect greater solar radiation than hair coats that are dark and dense or woolly. Furthermore, sheep with carpet wool protect themselves better from solar radiation by facilitating cutaneous heat dissipation (Sejian et al., 2018). Sheep with longer, thicker, and darker coats are subjected to greater stress and exhibit higher rectal temperature and sweating rates in tropical regions than white-haired sheep.

Goats are proficient desert-dwelling animals. Physiological characteristics of goats provides them an advantage over other ruminant species in harsh environmental conditions. Their small body size, fleece structure, and high digestive efficiency help them survive in harsh climatic conditions. Also, dwarf goats survive better in arid regions than other breeds, in part because their ears are short, erect and pointed forwards and their coat is a light color. Goats inhabiting arid zones possess long-hair, coarse-fiber fleeces to protect themselves from heat during the day and cold at night. Furthermore, goats in temperate areas have a coat of long coarse fibers and a seasonal coat of short, fine fibers to protect against extreme cold.

### Behavioral adaptation

Behavioral responses aid in the acclimatization process of animals when exposed to the high heat load. Behavioral responses studied in heat-stressed ruminants include shade seeking, reduced feed intake, increased water intake and drinking frequency, increased standing time, decreased lying time, and reduced defecation and urination frequency. Shade seeking is the most immediate behavioral response seen in heat-stressed animals. Typically, dairy cattle use shade on clear days once air temperature exceeds 21 °C, and the duration of shade use increases as air temperature and solar radiation increase, with cows often spending over 10 h per day under shade. Shade usage reduces grazing time and subsequently reduced milk production or reduced growth. Sheep, although typically more resilient, will also seek shade during exposure to elevated temperatures.

Reduced feed intake is an adaptive mechanism which reduces metabolic heat production in animals during summer. Numerous studies have reported reduced feed intake in cattle, sheep, and goats during exposure to heat (Valente et al., 2015; Aleena et al., 2018). Furthermore, Curtis et al. (2017) opined that behavioral studies showed variation in grazing patterns of extensively managed cattle under hot conditions with lower and higher grazing time during the day and night, respectively. Increased water consumption and drinking frequency occur in various ruminant livestock during hot conditions (Valente et al., 2015; Aleena et al., 2018). Brscic et al. (2007) established that heat-stressed cattle had a reduction in urination frequency while Chedid et al. (2014) reported that desert sheep compensate for the higher water loss by concentrating their urine during extreme heat load. Standing and lying time is also affected by high heat load. Heat-stressed sheep and cattle tend to spend more time standing to reorient themselves to avoid direct solar radiation and ground radiation.

### Physiological adaptation

Physiological adaptability is one of the primary response mechanisms that aids animal survival during exposure to high heat load. Exposure of animals to heat load induces an increase in the dissipation of excess body heat to the environment to reduce the heat load in their body. Further, dissipation of excess body heat is brought on by the physiological responses including increased respiration rate, rectal temperature, pulse rate, skin temperature, and sweating rate. Physiological responses show distinct diurnal variations during the daytime while the values remain stable during the night (da Silva et al., 2017). Reducing body heat at night helps the animals cope with higher temperature during the daytime. Respiration rate and rectal temperature are ideal indicators for quantifying heat stress in several ruminant species (Chauhan et al., 2014).


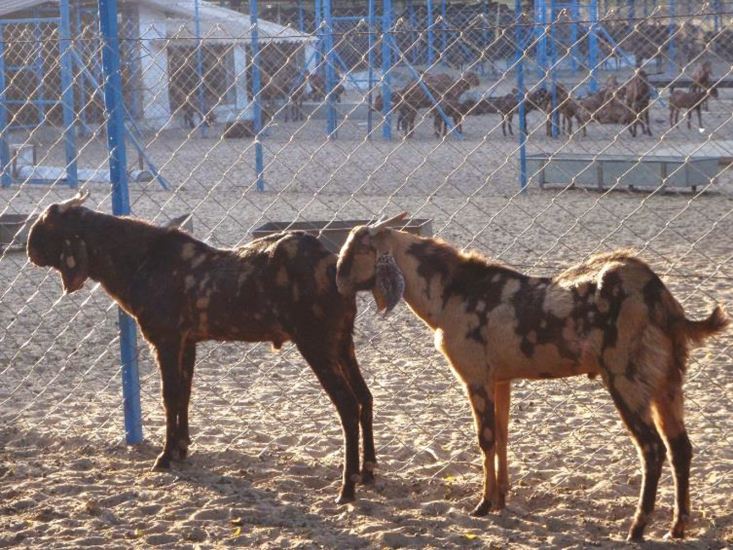


### Blood biochemical adaptation

Heat stress results in altered blood biochemical parameters. Heat stress induces an increase in hemoglobin and packed cell volume in cattle, and these changes are considered to be a gradual development of adaptive characteristics in cattle (Mazzullo et al., 2014). Furthermore, there are several hormones which are involved in controlling the mechanism of homeothermy in ruminant animals. In an effort to adapt with higher ambient temperatures, animals reduce the secretion of thyroid hormones to control metabolic activities and thus the production of body heat. Additionally, cortisol is the primary biochemical marker for heat stress in ruminant livestock. Substantial increases in levels of cortisol during heat stress indicates the stress level of ruminants (Sivakumar et al., 2010; Sejian et al., 2013). Superoxide dismutase and glutathione peroxidase are indicators of oxidative stress in sheep and cattle, particularly during exposure to excessive heat load. An increase in the concentration of these antioxidants was reported in sheep (Chandra and Aggarwal, 2009; Chaudhary et al., 2015) and dairy cattle (Bernabucci et al., 2002).

### Metabolic adaptation

When exposed to high heat load, the secretion of leptin and adiponectin are up-regulated, where leptin stimulates the hypothalamic axis resulting in a reduction in feed intake, while adiponectin changes the feeding behavior by peripheral and central mechanisms. Verma et al. (2000) attributed this decreased feed intake to the direct effect of increased temperature on the satiety center of the hypothalamus. Changes in the concentration of thyroid hormones in blood reflect the metabolic and nutrient status of the animal. The difference in the bioactivity of these hormones helps to maintain metabolic balance under stressful conditions, particularly in grazing animals since they are vulnerable to fluctuating environmental changes (Todini et al., 2007). It has been established in sheep that the decreased function of the thyroid gland during exposure to high heat load is a metabolic adaptation to reduce metabolic heat production. Increased ambient temperature can also directly affect the hypothalamic–pituitary axis and reduce thyroid stimulating hormone secretion. Decreased thyroid stimulating hormone production reduces thyroid gland function and circulating T3 and T4 hormones in an effort to reduce metabolic heat production. However, Chauhan et al. (2014) saw no change in cortisol, T3 or T4 in sheep exposed to excessive heat load.

Metabolic activities are also controlled by several enzymes. Plasma alkaline phosphatase and alanine aminotransferase concentrations generally increase in heat-stressed dairy cows. Serum alanine aminotransferase concentrations also increase in response to heat stress in sheep. The change in alanine aminotransferase and alkaline phosphatase during heat stress are indicators of poor liver function. Thus, both may be good markers in susceptible animals. Furthermore, nonesterified fatty acid also plays a crucial role in determining the energy status of livestock. Nonesterified fatty acids have a predominant role in maintaining metabolic activities through its timely mobilization to liver and peripheral tissues as a source of energy during periods of heat stress. Heat stress results in a considerable decline in nonesterified fatty acid concentrations in lactating cattle (Baumgard and Rhoads, 2013).

#### Biological markers.

Genetic differences in thermo-tolerance at the physiological and cellular levels in ruminant livestock have been well documented. Heat tolerance is a quantitative trait. One of the dominant genes identified to impart thermo-tolerance is the slick hair gene, which controls the length of hair in cattle. Apart from this, other genes such as ATPase Na+/K+ transporting subunit alpha 1 and ATPase Na+/K+ transporting subunit beta 2, thyroid hormone receptor, fibroblast growth factor, and heat shock proteins were found to be associated with heat tolerance in ruminants (Collier et al., 2012; Aleena et al., 2018). The ATPase Na+/K+ transporting subunit alpha 1 gene has also been associated with various heat tolerance variables including respiration rate and rectal temperature in both Tharparkar and Vrindavani cattle breeds suggesting that it may be a good biological marker for thermo-tolerance. Recently, researchers have established a rapid induction of heat shock protein-70 mRNA expression in goats during heat stress exposure confirming its role in heat tolerance (Aleena et al., 2018). In addition, polymorphisms in heat shock protein-90AA1 were also found to be associated with heat tolerance in Frieswal cattle (Deb et al., 2013) and sheep breeds (Marcos-Carcavilla et al., 2010). Increased expression of immune response genes such as a toll-like receptor, toll-like receptor 2/4 and interleukins 2/6 were also documented in heat stressed Tharparkar cattle. It is likely that these genes are associated with thermo-tolerance (Bharati et al., 2017). In a recent review, Sejian et al. (2018) identified respiration rate, rectal temperature, cortisol, plasma heat shock proteins-70, toll-like receptor-2, toll-like receptor-1, toll-like receptor-4, toll-like receptor-5, and heat shock proteins-70 genes to be useful biological markers for quantifying the impact of multiple stressors in both sheep and goats.

Knowledge of the impact of heat stress on the various adaptive responses provides clear insight into future ruminant livestock production. The various biological markers identified for the heat stress condition may also help researchers develop climate resilient breeds based on both phenotypic and genotypic markers involving morphological, behavioral, physiological, cellular, and molecular processes. In addition, combining the various identified biomarkers may help to look beyond thermo-tolerance in livestock and may go a long way to identify a breed or breeds with superior thermo-tolerance for optimum productivity. Therefore, with the advancement in assessing the various mechanisms associated with thermo-tolerance, it is possible to secure and sustain future ruminant livestock production by promoting welfare and favoring survival in a specific environment.

## Conclusions

Livestock are important contributors to total food production. Animal products are high-quality food, and they are an important source of income for many farmers in developing countries. Therefore, sustaining livestock production in a changing climate is one of the top priorities in the agriculture sector. Reducing the adverse impact of climate change on livestock requires multidisciplinary approaches including the integration of animal breeding, nutrition, housing, and health. It is essential to understand and analyze livestock responses to the environment, to design modifications of nutritional and environmental management strategies and thereby improve animal comfort and performance. However, in developing a strategy for adapting to climate change, one key challenge is dealing with uncertainty. Livestock producers should have key roles in determining the appropriate adaptation and mitigation strategies to use to sustain livestock production in a changing climate. The integration of new technologies into the research and technology transfer systems potentially offers many opportunities for further development of strategies to adapt to climate change.

## About the Authors

**John B. Gaughan** is an Associate Professor with a 25-year research career in the areas of livestock heat stress. He has authored or co-authored 12 book chapters, 44 refereed publications, 72 conference proceedings, and 20 research reports. He has been graduated 11 PhD and 3 MPhil students. He is recognized as a leader in cattle heat stress research in Australia and internationally. His research has helped to develop thermal stress models for feedlot cattle, dairy cows, and sheep. His works has focused on gaining an understanding of physiological responses to acute and chronic heat stress, the development of management and nutritional strategies to ameliorate heat stress, and the likely impact of future climatic conditions on animal production. John is also part of a team investigating nutritional strategies to ameliorate the effects of high heat load on feedlot cattle.

**Veerasamy Sejian, MVSc, PhD** is a Senior Scientist at ICAR-National Institute of Animal Nutrition and Physiology (NIANP), Bangalore, India. Dr Sejian is the team leader which established the concept of “Multiple stresses impacting small ruminant production in the changing climate scenario.” His current research is focused on identifying molecular markers for different environmental stresses in small ruminants with the primary focus to develop agro-ecological zone specific thermo-tolerant breed. Presently, he is running a Indo-French collaborative project “Emerging Crop-Livestock Production Systems adapted to a Changing Environment (ECLIPSE Project).” Further, Dr Sejian has published three international Springer books on collaborative mode—“Environmental stress and amelioration in livestock production”; “Climate change impact on livestock: adaptation and mitigation” and “Sheep production adapting to climate change.” For his outstanding contribution in climate change and livestock production, Indian Council of Agricultural Research (ICAR) has bestowed him with the prestigious Lal Bahadur Shastri Outstanding Young Scientist Award.

**Terry L. Mader** is Professor Emeritus at the University of Nebraska, Department of Animal Science. Dr Mader earned his BS from Kansas State University and his MS and PhD in Animal Nutrition from Oklahoma State University. Dr Mader’s current activities include involvement in national and international projects studying and mitigating environmental effects on animal productivity. He is past-Chairman of the FASS Beef Cattle subcommittee on the Guide for Care of Agricultural Animals and the Professional Animal Auditors Certification Organization, affiliated with American Registry of Professional Animal Scientists. Dr Mader is a previous recipient of the American Society of Animal Science Animal Management Award and an honorary professor of Animal Production at the University of Queensland in Australia. He is author or co-author of over 400 scientific publications. Dr Mader is currently Head of Mader Consulting, LLC, a business entity devoted to the development of management strategies and models to minimize stress and effects of climate variability in livestock.

**Frank Dunshea** is a Professor with a more than 30-year research career in the area of growth physiology and nutrition and the use of domestic animals in nutritional and biomedical research. He has published more than 750 journal, conference, book, patents, or technical articles. His research has had a high scientific impact, with many of the results being rapidly adopted by industry. Professor Dunshea is a respected research leader in global livestock industries and is committed to ensuring that livestock industries operate in a responsible and sustainable manner. Much of his work has focused on improving efficiency through reducing inputs and outputs while maintaining product quality and consumer health including an emphasis on mitigation against heat stress. In addition to many awards, he is a Fellow of the Australian Nutrition Society, the Australasian Pig Science Association, and the Australian Society of Animal Production as well as a former Chair of the Australian Academy of Science Committee for Nutrition.
